# Gas Chromatography Residue Analysis of Bifenthrin in Pears Treated with 2% Wettable Powder

**DOI:** 10.5487/TR.2009.25.1.041

**Published:** 2009-03-01

**Authors:** Jeong-Heui Choi, Xue Liu, Hee-Kwon Kim, Jae-Han Shim

**Affiliations:** 17 Natural Products Chemistry Laboratory, Division of Applied Bioscience and Biotechnology, College of Agriculture and Life Science, Chonnam National University, Gwangju, 500-757 Korea; 27 Vegetables Crops Experiment Station, Jeonnam Agricultural Research & Extention Service, Jeollanam-do, 542-821 Korea

**Keywords:** Bifenthrin, Wettable powder, Pear, GC-ECD

## Abstract

This study was conducted to monitor the level of bifenthrin residues in pear sprayed with 2% bifenthrin wettable powder (WP) at the recommended rate at four different schedules prior to harvest. The target analyte was extracted with acetone, partitioned into dichloromethane, and then purified by florisil chromatographic column. The residue determination was performed on a DB-5 capillary column using GC with electron capture detector (ECD). Linearity of this method was quite good (r^2^ = 0.9951) in the concentration ranged from 0.2 mg/kg to 10 mg/kg. Recovery test was carried out at two concentration levels, 0.2 mg/kg and 1.0 mg/kg, in three replicates, and their rates were from 82.9% to 107.2%. No quantitative bifenthrin was detected in pear of all kinds of treatments including the treatment sprayed 4 times until 7 days before harvest. This sensitive and selective method can be used to monitor the trace residual amounts of bifenthrin in pear in a quite low concentration level.

## INTRODUCTION

Nowadays, insecticides are commonly used for protecting pear production. Bifenthrin [2-methylbipenyl-3-ylmethyl (z)-(1RS, 3RS)-3-(chloro-3,3,3-trifluoroprop-1-enyl)-2,2-dimethylcyclopropanecarboxylate] is third-generation of the synthetic pyrethroids (SPs) family of insecticides (Fig. 1). This group has greater photostability and insecticidal activity than previous pyrethroids (Mokrey *et al.*, 1989). Bifenthrin is a non-cyano pyrethroid of strong and rapid insecticidal activity and low mammalian toxicity widely used in agricultural and public health applications (Barbara *et al.*, 2008; Miller *et al.*, 1985). Previous studies reported that enantioselective toxicity of SPs occurs in aquatic toxicity (Liu *et al.*, 2005; Wang *et al.*, 2007). Several studies have indicated that SPs could lead to oxidative damage in humans or animals which was associated with their toxic effects (Liu *et al.*, 2008). Some in vitro tests indicated that pyrethroids displayed cytotoxicity including DNA strand breaks and gene mutation (Majumder *et al.*, 1994; Surrallés *et al.*, 1995a, b). Pyrethroids had serious effects on fish and gill breathing aquatic insects

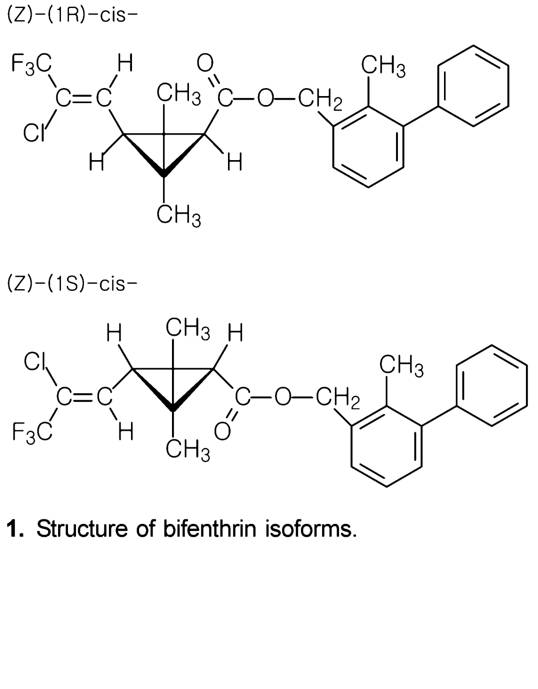

**Fig. 1.** Structure of bifenthrin isoforms.

because of the large surface area available to de-ionize after ATPase inhibition (Siegfried, 1993).

Bifenthrin was stable for 2 years at 25°C and 50°C (Tech.) (The pesticide manual, 12th edition). It has a low polarity and low solubility in water and a correspondingly strong tendency to bind to soil (Drenner *et al.*, 1992; Tomlin, 2000). Emily *et al.* studied occurrence and potential toxicity of pyrethroids and other insecticides in bed sediments of urban streams in central Texas, and found bifenthrin was the greatest contributor of insecticides in the sediments over the course of a year (Emily *et al.*, 2008). There were also several other reports suggested that the pyrethroids played a dominant role in the toxicity of urban creeks in California (Weston *et al.*, 2006; Amweg and Weston, 2007; Weston and Amweg, 2007). Bifenthrin could be potentially adsorbed on the pear after spraying. So it's necessary to monitor the residue amounts prior to consumption. The determination of bifenthrin has been mainly reported by gas chromatography (GC) (Liu *et al.*, 2004; Rosa *et al.*, 2008; Shen, 2002; Zeng *et al.*, 2008), high performance liquid chromatography (HPLC) (Wei *et al.*, 2006) and enzyme-linked immunosorbent assay (ELISA) (Li *et al.*, 2008). The purpose of this investigation was to regularly monitor the bifenthrin residues in pear through analytical method that combined short analysis time, sufficient selectivity and sensitivity.

## MATERIALS AND METHODS

***Solvents and reagents.*** Analytical standard of bifenthrin (95%) was supplied by Dow AgroSciences (Seoul, Republic of Korea). Acetone, *n*-hexane, dichloromethane (HPLC grade) and sodium sulfate anhydrous (GR grade) were purchased from Merck (Darmstadt, Germany). Celite 545 (analytical grade) was from Duksan pure chemicals Co., Ltd. (Korea). Florisil (60~100 mesh, pesticide residue analytical grade) was purchased from Sigma (Missouri, USA).

***Apparatus.*** A Daihan scientific wise Mix™ HG-150 (Korea) homogenizer was used for sample homogenization. A rotary evaporator (Büchi Rotavapor R-114, Switzerland) was used to evaporate extract under vacuum condition. A HP 5890 Series II gas chromatography (Hewlett Packard, USA) with electron capture detector (ECD) was used to analyze bifenthrin residues in pear. Separation was performed on a DB-5 capillary column (30 m × 0.53 mm i.d, 0.50 μm film thickness). Injection and detector temperature was 270°C and 300°C, respectively. Injection volume was 1 μl. Nitrogen gas with a constant flow of 5.0 ml/min was applied as a carrier gas. The temperature program was started at 250°C then increased at the rate of 2°C/min to 265°C, maintained for 2.5 min.

**Table 1.** Spraying schedule of bifenthrin formula on pear trees

| The number of treatments | Spraying date prior to harvest | Final date of spraying | Concentration of pesticide formula |
|---|---|---|---|
| 0 | -- | No treatment | |
| 2 | 40-30 | 9/2 | 500 l of dilution |
| 3 | 40-30-21 | 9/11 | /10a, |
| 4 | 40-30-21-14 | 9/18 | (a.i 0.01 kg/10a) |
| 4 | 30-21-14-7 | 9/25 | |

***Treatment design and sampling.*** We made 5 groups with different treatment of 2% bifenthrin WP. One group with no treatment was kept as a control. The other groups were separately sprayed with 1000 times dilution liquid of 2% WP for 2, 3 and 4 times in different days before harvest. The details were shown in Table 1. Each group was repeated in three times by making same three plots divided.. We collected 2 kg sample in each group on October second, 2005. The weight of one pear was 348.4 g ± 10.5 g (n = 30). The collected pears were chopped and mixed well and kept in refrigerator at -70°C before experiment.

***Preparation of standard solution.*** Stock standard solution was prepared by dissolving 10.526 mg of bifenthrin standard (95%) in 100 ml acetone (100 mg/l). Then, a series of working solutions with the concentration of 0.2, 0.5, 1, 2, 5, 10 mg/kg were prepared for calibration curve.

***Sample preparation.*** A portion of 20 g of pear was homogenized with 100 ml of acetone at 1100 rpm for 2 minutes. The content was filtered by filter paper (Whatman No. 6) and 20 g of celite with the support of büchner funnel. The extract was transferred into a 500 ml-separatory funnel. Fifty milliliter of saturated sodium chloride aqueous solution and 200 ml of distilled water were added. Then, 50 ml of dichloromethane was added for partitioning extraction of the target compound. The dichloromethane extract was dehydrated by sodium sulfate anhydrous. Another 50 ml of dichloromethane was used to extract again. The dichloromethane extract were combined and evaporated at 40°C. The residue was reconstituted in 10 ml of *n*-hexane for cleanup using open preparative chromatographic column (1.1 cm, i.d.) packed with 10 g of florisil. The column was washed with 50 ml of *n*-hexane/dichloromethane (9 : 1, v/v) and eluted with 120 ml of dichloromethane/*n*-hexane/acetonitile (50 : 49.65 : 0.35, v/v). The eluate was evaporated and reconstituted in 2 ml of *n*-hexane, then 1 μl of samples was injected into GC-ECD.

## RESULTS AND DISCUSSION

***Linearity.*** The detector responses and the amounts of bifenthrin investigated (6 points) were well linear with a correlation coefficient of $r^2 = 0.9951$, slope of 1621.8 and y-intercept of 756.38 in the range from 0.2 to 10 ng.

***Recovery.*** The recovery experiment was carried out by spiking bifenthrin into blank pear at 2 concentration levels in triplicate. The recoveries ranged from 82.9 to 107.2%. The limit of detection (LOD) was 0.02 mg/kg. The minimum detection level (MDL) of analytical instrument was 0.2 ng (Table 2). The LOD was much lower than the maximum residue limits (MRL, 0.5 mg/kg), according to the Korea Food & Drug Administration (KFDA, 2005).

The described method for the analysis of bifenthrin residues in pear sample was quite sensitive and selective. The detection time was less than 10 minutes in a running cycle. There was no interruption materials could be detected in the same retention time with bifenthrin standard (Fig. 2).

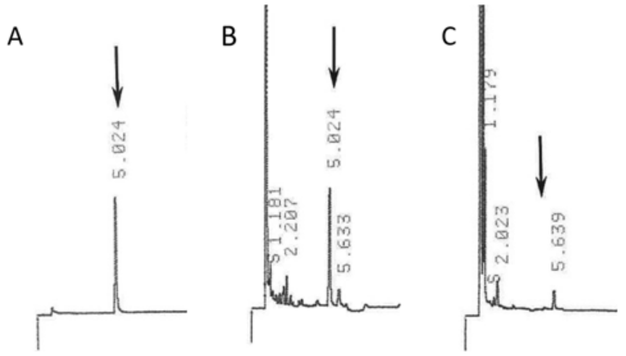

**Fig. 2.** Representative chromatograms of (A) bifenthrin standard, (B) fortified pear sample at 0.2 mg/kg and (C) blank.

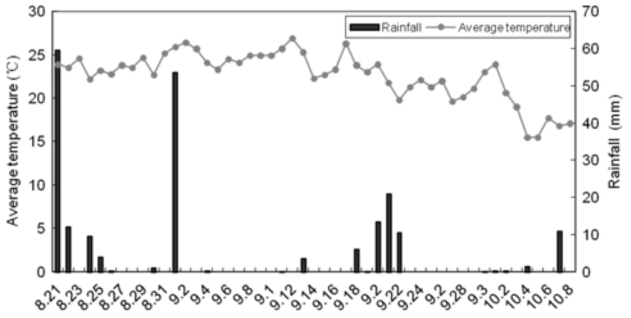

**Fig. 3.** Weather information (temperature and rainfall) during field experiments.

***Analysis of field samples.*** We analyzed the bifenthrin residues in pear of these five groups in triplicate. No quantitative bifenthrin was detected in all of sample (Table 3). Even in fifth group, we sprayed 4 times and the latest sprayed date was only 7 days before harvest, the bifenthrin residual concentration was lower than LOD. We recorded the weather information during the investigation (Fig. 3). The arrows indicate the spraying date. The absence of bifenthrin residues may be caused by the rainfall, decomposition or the other complex conditions even though we always sprayed until 4 hours later after rained. Weston reported that stormwater runoff was very important in transporting pyrethroids to urban creeks (Weston *et al.*, 2008). It may

**Table 2.** Recovery, limit of detection (LOD) and minimum detection level (MDL)

| Sample | Fortified concentration (mg/kg) | Recovery (%) | | | | LOD (mg/kg) | MDL (ng) |
|---|---|---|---|---|---|---|---|
| | | 1 | 2 | 3 | mean | | |
| Pear | 0.2 | 107.2 | 105.0 | 106.4 | 106.2 ± 1.1 | 0.02 | 0.2 |
| | 1.0 | 92.5 | 87.1 | 82.9 | 97.5 ± 4.8 | | |

**Table 3.** Bifenthrin residual concentrations in pear

| The number of treatments | Spraying date prior to harvest | Bifenthrin residual concentration (mg/kg) | | | MRL (mg/kg) |
|---|---|---|---|---|---|
| | | 1 | 2 | 3 | |
| No treatment | -- | < 0.02[a] | < 0.02 | < 0.02 | 0.5 (Pear, KFDA) |
| 2 | 40-30 | < 0.02 | < 0.02 | < 0.02 | |
| 3 | 40-30-21 | < 0.02 | < 0.02 | < 0.02 | |
| 4 | 40-30-21-14 | < 0.02 | < 0.02 | < 0.02 | |
| 4 | 30-21-14-7 | < 0.02 | < 0.02 | < 0.02 | |

[a]Limit of detection indirectly explain the disappearance of bifenthrin in the pear. As shown in Fig. 3, there were plenty of precipitations during spraying schedule. It can be deduced that much bifenthin may washed away by rain. But the spraying were carried out under real weather conditions. And the results indicated that all of the four different spraying schedules were acceptable for the application of bifenthrin in pear before harvest. No official spraying program of bifenthrin in pear has been established in Korea. So, our researches can give a preliminary support for the usage of 2% WP bifenthrin in pear.

There are a good many pesticides used for protecting pear samples, such as amitraz, chlorfluazuron, fenthion haptens, pyrethroids and so on. Amitraz and its four degradation products were detected by LC with quadrupole time-of-flight mass spectrometry (QqTOF-MS and MS/MS) in pear samples (Pico *et al.*, 2008). Chlorfluazuron could be monitored with HPLC-UVD, which was also our previous work (Shim *et al.*, 2007). Fenthion haptens determination method was developed by Zhang with a sensitive ELISA method (Zhang *et al.*, 2008). In this work, the bifenthrin which is extensively used due to its good performance among pesticides was studied by a simple, high sensitivity and adaptable to common laboratory method for its determination in pear sample. Our experimental method using traditional extraction and cleanup way and general GC-ECD apparatus is able to obtain high sensitivity and reliability for the determination of bifenthrin in pear sample.

## CONCLUSION

The experiment of residue analysis of 2% bifenthrin wettable powder in pear by GC-ECD was successfully accomplished. The method was sensitive and selective for determination of bifenthrin in pear. No bifenthrin was detected in each group sample of different sprayed program. It is safe to use 2% bifenthrin wettable powder 4 times until 7 days before harvest to protect pear against insects under experimental conditions.

## ACKNOWLEDGEMENT

This study was mainly supported by Dow AgroScience in Republic of Korea.
